# Scaling-up implementation in community hospitals: a multisite interrupted time series design of the Mobilization of Vulnerable Elders (MOVE) program in Alberta

**DOI:** 10.1186/s12877-019-1311-z

**Published:** 2019-10-25

**Authors:** Jayna Holroyd-Leduc, Charmalee Harris, Jemila S. Hamid, Joycelyne E. Ewusie, Jacquelyn Quirk, Karen Osiowy, Julia E. Moore, Sobia Khan, Barbara Liu, Sharon E. Straus, on behalf of the MOVE AB Collaboration, Colin Zieber, Dianne Nolette, Gloria Nemecek, Godfrey Quan, Jeff Garett, Jennipher Martin, Joel Weaver, Joyce Nay, Kerry Roberts, Leslee Remmie, Lynn Goughnour, Teri Myhre, Brenda Ashman, Byron Hirsch, Erin Stodalka, Linda Iwasiw, Pat Marshall, Racquel Charnetski, Robert J. Hiobbard, Sharlene Krein, Sheila Burkart, Myra Gerhardt, Audrey McKenzie, Brenda Bond, Leanne Haines-Doig, Lori Sparrow, Mollie Cole, Rupinder Brar, Ruksana Rashid, Steven Turner, Tracey Black, Wayne Krejci, Beth Mulat, Brenda Clark, Caitlan Reich, Cindy Belanger, Danuta Kolodziej, Jessica Best, Jill Chow, Jody Prohar, Kimberly Henry, Kristine Hayday, Jennifer Stickney Lee, Michelle Kitsco, Rebecca Curran, Sandra Mageau, Shirley Baumgartner, Treena Hinse, Wendy Tanaka

**Affiliations:** 1 Departments of Medicine and Community Health Sciences, Cumming School of Medicine, University of Calgary, Calgary, Canada; 2 Specialized Geriatric Services, Alberta Health Services, Calgary, Canada; 3 Li Ka Shing Knowledge Institute, St. Michael’s Hospital, Toronto, Ontario Canada; 4 Department of Health Research Methods, Evidence, and Impact, McMaster University, Hamilton, ON Canada; 5 Children’s Hospital of Eastern Ontario, Ottawa, ON Canada; 6 School of Epidemiology and Public Health, University of Ottawa, Ottawa, ON Canada; 7 Public Health Ontario, Toronto, Ontario Canada; 8 Regional Geriatric Program of Toronto and Sunnybrook Health Sciences Centre, Toronto, Ontario Canada; 9 Department of Medicine, Faculty of Medicine, University of Toronto, Toronto, Ontario Canada

**Keywords:** Mobilization, MOVE, mobility, older adults, scale and spread

## Abstract

**Background:**

As the population ages, older hospitalized patients are at increased risk for hospital-acquired morbidity. The Mobilization of Vulnerable Elders (MOVE) program is an evidence-informed early mobilization intervention that was previously evaluated in Ontario, Canada. The program was effective at improving mobilization rates and decreasing length of stay in academic hospitals. The aim of this study was to scale-up the program and conduct a replication study evaluating the impact of the evidence-informed mobilization intervention on various units in community hospitals within a different Canadian province.

**Methods:**

The MOVE program was tailored to the local context at four community hospitals in Alberta, Canada. The study population was patients aged 65 years and older who were admitted to medicine, surgery, rehabilitation and intensive care units between July 2015 and July 2016. The primary outcome was patient mobilization measured by conducting visual audits twice a week, three times a day. The secondary outcomes included hospital length of stay obtained from hospital administrative data, and perceptions of the intervention assessed through a qualitative assessment. Using an interrupted time series design, the intervention was evaluated over three time periods (pre-intervention, during, and post-intervention).

**Results:**

A total of 3601 patients [mean age 80.1 years (SD = 8.4 years)] were included in the overall analysis. There was a significant increase in mobilization at the end of the intervention period compared to pre-intervention, with 6% more patients out of bed (95% confidence interval (CI) 1, 11; *p*-value = 0.0173). A decreasing trend in median length of stay was observed, where patients on average stayed an estimated 3.59 fewer days (95%CI -15.06, 7.88) during the intervention compared to pre-intervention period.

**Conclusions:**

MOVE is a low-cost, effective and adaptable intervention that improves mobilization in older hospitalized patients. This intervention has been replicated and scaled up across various units and hospital settings.

## Background

A demographic shift is occurring in several countries, including Canada, with those over 65 years of age accounting for an increasing proportion of the population [1]. Many of these older adults are frail and/or have chronic diseases, which can contribute to acute care hospitalization [2, 3]. When hospitalized, older patients experience higher rates of adverse events and complications than younger patients [4]. The consequences to the individual and the health care system can include increased risk of delirium, loss of the ability to live independently, increased length of hospital stay, and hospital readmission [4–6]. One-third of older adults develop a new disability in an activity of daily living during hospitalization and half will not recover function [6, 7].

Functional decline in hospital can be prevented by early mobilization [8–11]. However, currently hospitalized older adults spend most of their stay in bed [12, 13]. Multidisciplinary exercise interventions targeted towards older patients have been found to significantly increase hospital discharges to home, decrease length of stay and decrease hospital costs [14]. The benefits of early mobilization have been demonstrated among a variety of patient populations [9–11, 15]. The broad implementation of an interprofessional pragmatic early mobilization program within academic hospitals across Ontario, Canada, entitled Mobilization of Vulnerable Elders (MOVE), increased mobilization rates and decreased hospital length of stay among older adults [16, 17]. This program was tailored to the local context and shifts the idea that mobilization is a task designated to a single professional group, typically physical therapists, to a shared responsibility among all members of the healthcare team. The objective of the current study was to determine if MOVE could be successfully replicated, scaled-up, implemented and sustained within non-academic hospitals in another Canadian province. The specific objectives were to scale-up and spread MOVE to four community hospitals across Alberta, Canada.

## Methods

Aligning with the original MOVE study design, we used a pragmatic, quasi-experimental un-blinded interrupted time series (ITS) design to evaluate the effectiveness of the intervention. An ITS design is used to evaluate the effectiveness of an intervention by using a continuous sequence of observations in which an intervention is implemented at a defined point in time [18]. The primary outcome of the analysis was rate of mobilization collected over three time periods: pre-intervention (10 weeks), during intervention (8 weeks) and post-intervention (20 weeks). We completed the Standards for Quality Improvement Reporting Excellence (SQUIRE) guidelines and the Template for Intervention Description and Replication (TIDieR) checklist [Additional file 1]. Details of the original study methods were described in a protocol paper [17]. The protocol was approved by the Conjoint Health Research Ethics Board (CHREB) at the University of Calgary (REB# 14-479), Calgary, Alberta, Canada.

### Setting and participants

The MOVE AB intervention was conducted on seven units at four community hospitals in Alberta, Canada. Units included: one long-term/restorative care, one general medicine, one surgical, one geriatric rehabilitation, one short stay surgical and two intensive care units. The participating units were selected by local leadership and the MOVE program was conducted between July 2015- July 2016. Patients included in the study were aged 65 years and older who were admitted to participating units. Patients receiving palliative care or on bed rest were excluded.

#### Demographics

Demographic data of patient characteristics (i.e., age, gender, admitting or most responsible diagnosis, discharge destination) were summarized overall, and for the three different time periods. Demographic data was analyzed and presented using descriptive statistics. Patient diagnoses were coded using the International Classification of Diseases version 10 (ICD-10) codes.

### Study design

#### Description of the intervention

The Mobilization of Vulnerable Elders (MOVE) intervention is an interprofessional approach that focuses on early and consistent mobilization of older adults admitted to hospital. The intervention focuses on implementing three key messages into practice: 1) patients should be assessed for mobilization status within 24 hours of admission; 2) mobilization should occur at least three times a day; and 3) mobility should be progressive and scaled.

To implement these three key messages, MOVE consists of three core program components: 1) an education component in which the three key messages must be conveyed; 2) a patient/family member component to focus on teaching patients and their family members about the benefits of early mobilization, and; 3) a sustainability component, to ensure that site implementation teams are considering sustainability early in the planning process. To ensure the intervention is adapted and tailored to local context, sites also selected appropriate implementation activities based on their context.

Local implementation teams were created at each site to help facilitate core program components, such as assessment and planning, and to promote the intervention. Each site had approximately 4-5 local implementation team members that consisted of a physician lead, research coordinator, nursing education coordinator and other key staff members such as allied health professionals. A centralized team of implementation support specialists provided ongoing assistance to ensure the intervention was implemented effectively. Steering committee meetings were also held on a monthly basis for the team members from the four participating sites to engage in ongoing dialogue and discuss experiences and lessons learned with other sites throughout the project. These committee meeting were also attended by members of the central team.

### Implementation process

The implementation process was guided by the knowledge to action (KTA) cycle [19]. Each site began the MOVE AB intervention in the first week of July 2015 (see Additional file 2 for MOVE program timeline). The first 10 weeks of the study was pre-intervention phase, in which a readiness assessment was employed [20–22]. Each participating unit completed a readiness assessment survey to understand organizational readiness for implementation. Readiness assessments were administered to staff at the unit level. As part of a sub-study, a cluster randomized trial was employed to compare the effects of using a decision support tool to select a readiness measure and selecting a readiness measure without the aid of a decision tool (findings not presented here). Two randomly selected sites selected their own readiness assessment measure from a list of validated readiness measures and the remaining two sites used the Ready, Set, Change! Decision Support tool (http://readiness.knowledgetranslation.ca), a decision aid tool that selects the most appropriate readiness measure based on the organizational setting [20]. Implementation activities were selected using an online barriers and facilitators assessment tool, the Select, Tailor, Plan and Engage (STEP) tool informed by the theoretical domains framework [23–25]. This online tool was completed by participating units to determine areas of improvement and to help inform the selection and development of appropriate implementation activities at their site, thus considering local context. Sites accessed all tools and resources using the MOVE online portal through the MOVE website (www.movescanada.ca). We monitored implementation activities using an implementation process tool.

To plan for sustainability, site implementation teams completed the NHS Sustainability Survey in both the pre-intervention and post-intervention phases [26]. The purpose of the pre-intervention NHS Sustainability Survey was to determine the overall climate of sustainability and what strategies should be implemented as part of the sustainability component of the intervention. The purpose of the post-intervention NHS Sustainability Survey was to determine whether there were changes in the climate of sustainability throughout the intervention and whether additional considerations for sustainability were necessary. The NHS Sustainability findings were disseminated to sites and utilized as an implementation support tool (Additional file 3).

### Measures

Outcome ascertainment was consistent across sites. The primary outcome was the proportion of patients aged 65 and older who were mobilized at least once daily per project week, where visual audits were conducted three times per day for two days each week during the pre-intervention phase (10 weeks), intervention phase (8 weeks), and post-intervention phase (20 weeks). Eligible patients were identified and observed over time using unit -specific administrative data (e.g., daily patient census report) prior to conducting audits. Patient identification numbers and types of mobility observed were recorded using an audit tool (Additional file 4). Types of mobilization considered in the analysis included standing/walking with assistance, standing/walking supervised, and standing/walking independently. Immobile patients were those lying in bed, sitting in bed in upright position, sitting on a chair, and those who could not be observed (to allow a conservative estimate of rate of mobilization). Observational patient audits were documented by a trained research assistant at each participating site [17]. This method of mobility audit was previously compared to continuous rounding (positive likelihood ratio, 12.2; negative likelihood ratio, 0.06) [16]

Secondary outcomes were hospital length of stay (LOS) and perceptions of the intervention from site implementation teams, participating unit staff and patients/family members. LOS data were obtained from hospital administrative data along with age, gender, patient location prior to admission, and admitting diagnosis of eligible patients. Perceptions of unit staff on the involved hospital units, and of patients and their family members were collected through anonymized exit surveys (Additional file 5 and Additional file 6). In addition, the central team conducted semi-structured interviews with site implementation team members to explore their perception of the intervention and suggestions for facilitating sustainability. Implementation teams were invited to participate in interviews via email by the MOVE implementation coach. Each interview was approximately 60 minutes in length and conducted over the phone by a member of the MOVE Central Team.

### Statistical analysis

#### Primary outcome

The primary outcome was the proportion of patients aged 65 and older who were mobilized (out of bed) at least once daily per project week (3 audits/ day; 2 audit days per project week). The proportion of patients mobilized was averaged over the two audit days to provide an estimate of average daily mobilization for a given week. This primary analysis was done pre- (10 weeks, 20 assessment points), during (eight weeks, 16 assessment points), and post-intervention (20 weeks, 40 assessment points) at each hospital. Mobilization at each time point was aggregated across all participating sites to provide an overall estimate of daily mobility. Overall impact of the intervention was examined through an ITS analysis using a segmented linear regression model [27]. Presence of serial autocorrelation across the different time points was assessed using the Durbin-Watson’s statistic [28]; and when statistically significant, adjustment for autocorrelation was performed [29].

### Secondary outcomes

#### Length of stay

Patients audited for mobilization in the pre-, during- and post-intervention implementation were included in the analysis. Median daily LOS was calculated for each of the weeks in the study period, classified into the different periods (pre-, during and post-intervention implementation). Similar to the mobilization outcome, segmented regression was used to investigate and compare the trend in median LOS among the three time periods. We performed overall (across all sites) and site-level analyses.

All statistical analyses were performed using the R statistical package. Statistical significance was determined at α=0.05 level of significance.

#### Staff and patient/family member exit surveys and implementation team interviews

Quantitative data from staff and patient/family member exit surveys were analyzed using descriptive statistics. Interview data was independently analyzed by two coordinators on the central implementation team using a thematic analysis approach [30]. First, the analysts familiarized themselves with the data and past MOVE projects to develop initial coding themes, which were then further refined to develop the coding framework. This framework was then used to analyze a sample of transcripts, and then any modifications were made to the framework after discussion between the two analysts. Lastly, all transcripts were coded by the analysts independently, using the final coding framework. Inter-rater reliability (i.e., the degree of agreement between two coders) was compared through the calculation of percentage agreement, and any discrepancies (defined as less than 80% agreement) were reconciled through discussion after the familiarization stage. All analysis was conducted using NVivo 11 software.

## Results

### Demographics

A total of 42,840 mobility audits from 3,601 unique patients were completed within the four participating community hospitals in Alberta, Canada [Table 1]. These hospitals provide acute care services to communities ranging in size between 9,000 and 90,000 individuals. The seven participating units across the four hospitals varied and included surgery, geriatric rehabilitation, general medicine, intensive care, and long term/restorative care. The mean age of the included patients was 80.1 years (Standard deviation [SD] = 8.4 years) across the four sites. The most common diagnoses by patient visit were chronic obstructive pulmonary disease, heart failure, urinary system disorders, pneumonia, and ileus/intestinal obstruction.

**Table 1:** Demographic and clinical characteristics of MOVE subjects stratified by age, gender, most responsible discharge diagnoses, location prior to admission, and discharge destination

		Intervention Phases			
		Overall *n* = (4036)	Pre *n* = (1054)	During *n* = (654)	Post *n* = (2328)
No. of unique subjects, N		3601	890	569	2142
Age^a^, mean (sd)		79.90 (8.585)	79.84 (8.435)	79.89 (8.912)	79.93 (8.562)
Gender^a^ M: F, n (%)		1733 (48.1): 1868 (51.9)	435 (48.9): 455 (51.1)	284 (49.9): 285 (50.1)	1014 (47.3): 1128 (52.7)
Top 5 Most Responsible Discharge Diagnoses, n (%)	Chronic Obstructive Pulmonary Disease	278 (6.9)	68 (6.5)	50 (7.6)	160 (6.9)
	Heart Failure	253 (6.3)	60 (5.7)	35 (5.4)	158 (6.8)
	Urinary System Disorders	126 (3.1)	34 (3.2)	20 (3.1)	72 (3.1)
	Pneumonia	113 (2.8)	29 (2.8)	15 (2.3)	69 (3.0)
	Paralytic Ileus and Intestinal Obstruction Without Hernia	105 (2.6)	24 (2.3)	21 (3.2)	60 (2.6)
Location prior to admission, n (%)	Private home, apartment or condominium	521 (12.9)	132 (12.5)	76 (11.6)	313 (13.4)
	Another acute facility	423 (10.5)	100 (9.5)	74 (11.3)	249 (10.7)
	Nursing home or LTCH	24 (0.6)	5 (0.5)	4 (0.6)	15 (0.6)
	Rehab	–	–	–	–
	Other	3068 (76.0)	817 (77.5)	500 (76.5)	1751 (75.2)
Discharge Destination	Home	2846 (70.5)	763 (72.4)	488 (74.6)	1595 (68.5)
	Rehab	–	–	–	–
	Nursing Home	535 (13.3)	124 (11.8)	69 (10.6)	342 (14.7)
	Acute Facility	270 (6.7)	82 (7.8)	40 (6.1)	148 (6.4)
	Deceased	309 (7.7)	70 (6.6)	43 (6.6)	196 (8.4)
	Other	76 (1.9)	15 (1.4)	14 (2.1)	47 (2.0)

^a^Based on number of unique subjects

### Implementation strategies

Each unit completed the STEP Tool, which provides sites with a list of strategies that implementation teams could choose from based on each site’s identified barriers and facilitators. Each site was unique in terms of their overall patient, provider, setting, and implementation barriers. Sites were able to prioritize key barriers (e.g., patient/family beliefs about mobilization, presence of other unit priorities) and select appropriate strategies. There were similarities and differences in terms of barriers and chosen strategies between and across sites. All sites chose to use champions and documentation (i.e., use of whiteboards, log sheet), in addition to the mandatory components of educational meetings and audit-feedback. Sites were able to tailor materials to the local context, for example they could use local logos and embed MOVE key messages into existing program materials. For example, a surgical unit incorporated MOVE strategies into their recovery patient materials. Other commonly utilized strategies included reminders and educational materials. All sites also had patient education activities (e.g. bedside education) and educational materials (e.g. pamphlets, posters). For a complete list of selected implementation strategies, please see Additional file 7.

### Mobilization rates

**Fig. 1:**
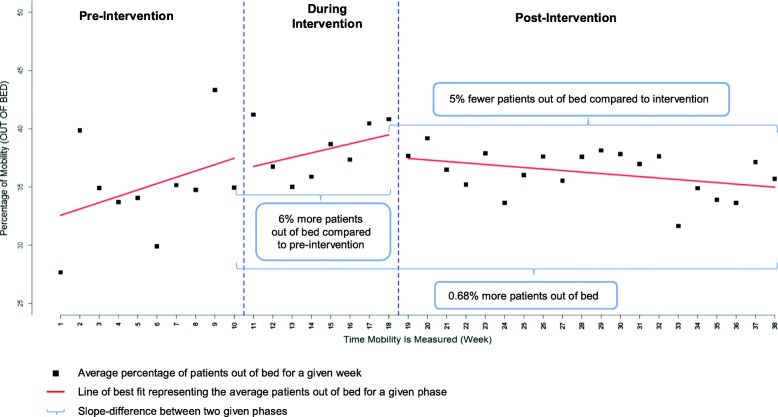
Interrupted time series analysis representing overall weekly visual audit results for proportion of patients out of bed over 38 weeks for all 4 participating sites

There are an estimated 6% (95% CI: [1, 11], *p*-value=0.0173) more out of bed audits at the end of the intervention period compared to end of pre-intervention period (Fig. 1). A decrease in rate of mobilization was observed post intervention implementation, where a slope difference of -0.68% (95% CI [-1.17, -0.18], *p =* 0.0073) was estimated between pre- and post-intervention implementation. Consequently, an estimated -5% (95% CI: [-10, -0.1], *p*-value=0.0443) fewer out of bed daily audits were observed at the end of the post-implementation period compared to during intervention.

### Length of hospital stay

**Fig. 2:**
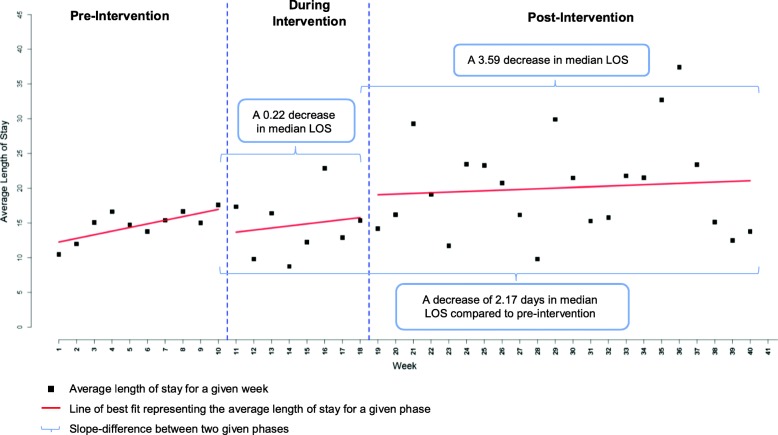
Interrupted time series analysis comparing median hospital length of stay between pre-, during and post-intervention for all 4 participating sites

During the intervention period, there was a non-statistically significant slight decrease in median LOS observed, where patients on average stayed an estimated 3.59 fewer days (95% CI:[− 15.06, 7.88], *p*-value = 0.5401) in hospital compared to post-intervention implementation (Fig. 2). An increase in median LOS was observed immediately post-intervention followed by a slight increasing trend. Patients stayed 2.17 (95%CI:[− 20.49, 16.15], p = 0.8162) less days in hospital post-intervention implementation compared to the pre-intervention implementation.

### Perceptions of the MOVE intervention

Exit surveys were completed with 29 staff members (8 nurses; 5 physicians; 2 administrators; 2 physiotherapists; 11 other allied health professionals) and 45 patients (39 patients; 3 family members/caregivers; 3 unidentified). For staff and patient participants stratified by site, please see Additional file 8.

Most staff perceived the educational activities as effective in increasing staff knowledge on early mobilization. Specifically, survey respondents perceived staff education (72%; *n* = 21) and educational materials (76%; *n* = 22) as being effective in increasing their knowledge. A total of 48% (*n* = 14) of staff respondents believed that patient and family members’ knowledge about early mobilization increased. Similarly, 48% (*n* = 14) of staff respondents perceived patient and family members support for early mobilization increased as a result of MOVE.

One of the goals of MOVE is to encourage an interprofessional approach to mobilizing patients, where all staff share in the responsibility. A total of 41% (*n* = 12) of staff respondents believed that there was a change in staff roles and responsibilities for mobilization over the course of MOVE.

A total of 51% (*n* = 23) of patients believed education activities and materials were effective in increasing their knowledge about the importance of moving during their hospital stay. Although fewer patients (40%; *n* = 18) perceived the educational activities to be effective in helping them move during their hospital stay, 59% (*n* = 24) identified that staff always or frequently encouraged them to perform more mobilization activities.

### Implementation and sustainability

Nine site implementation team members representing all four sites participated in interviews to provide feedback on their implementation experience with MOVE. See Additional file 9 for participant breakdown by profession and site. Keeping staff abreast of MOVE activities, outlining the intended purpose of each step, and providing updates were all ways identified to help support staff participation. Most sites held implementation planning meetings. Involving front-line staff, especially champions, in the planning and implementation activities was seen as a key strategy and helpful at all sites.

Interview participants identified that education sessions with staff were a key strategy to do early on to disseminate information and engage frontline staff. Regular, dedicated time with unit staff to discuss MOVE implementation was perceived to be important for making staff aware of the intervention, and was an opportunity to identify complementary initiatives to support mobilization with both staff and patients. Interview participants expressed the overall importance of embedding the practice change into work processes and organizational culture, so that MOVE no longer felt like a program or new initiative.

Consistent communication between the central coaches and the sites was also seen as an effective way of providing ongoing support. Steering committee meetings gave sites the opportunity to share and learn from each other, including discussing common challenges and potential solutions. Interview participants also felt that the feedback they received on intervention progress throughout implementation was helpful.

All sites (*n* = 4) completed the NHS Sustainability survey during pre- and post-intervention to identify strengths and weaknesses in implementation planning and predict the likelihood of sustainability (Additional file 3). Based on the NHS Sustainability Guide, an overall score of 55 or higher suggests that a site is likely on track for sustainability, a score between 45 and 55 suggests making small changes to get on track for sustainability, while a score of 45 or lower recommends a site create a sustainability plan to increase the likelihood initiative will sustain. Based on pre-intervention survey results, two sites, Site B and Site C had a sustainability score above 55 (62.9 and 85.1), Site A had a sustainability climate score of 45.2 (between 45 and 55), while Site D had a score of 23 (45 or lower). Common strengths from sites were strong senior leadership engagement and adaptability of sites in facing ongoing changes in staff, leadership, organization structures etc. Challenges during pre-intervention were lack of infrastructure to enable sustainability (e.g., policies and procedures to reflect new processes) and lack of staff involvement and training to sustain the process. During post –intervention, sites with lower sustainability scores demonstrated an increase in sustainability climate where Site D had a sustainability climate score of 38.6 and Site A had a sustainability score of 62.9. Site B demonstrated a small decrease in sustainability with a score of 60.6 whereas Site C had a substantiality score of 40.9. During post-intervention, a common strength amongst sites was clinical leadership engagement and a common challenge identified was lack of senior leadership engagement.

## Discussion

The four community hospitals participating in MOVE demonstrated an increase in the number of patients out of bed with the intervention and a possible trend towards decreased hospital length of stay. These results are comparable to the increase in mobilization rates and decrease in hospital length of stay previously observed in 14 academic hospitals [16]. This indicates that we were able to scale up an evidence-informed program, while maintainting it’s effectiveness [31]. A systematic tailored approach that targets locally identified barriers and facilitators, as well as underlying behaviour change theory, is an effective approach in selecting the appropriate staff, patient, and organiational level implementation strategies to promote early mobilization [32, 33].

The results align with studies performed in other settings across various clinical settings including critical care units, and medical and surgical inpatient units [34–39]. These previous studies have typically focused on implementation across a single site. They have also highlighted various approaches to enhancing mobility including those led by various team members including nurses and rehabilitation therapists. MOVE is unique in is team-based approach to enhancing mobility as well as the focus on enhancing mobility across different types of inpatient settings. Other unique features of MOVE, which contrast with these other studies, include that the funding model of MOVE focused on funding the evaluation, printed resource materials and centralized implementation coaching services. Thus, MOVE is an intervention that can be implemented and sustained without the requirement for new clincial resources. We effectively utilized existing clincial resources and considered the local context and barriers. Additionally, this study demonstrated that this intervention can be spread beyond urban academic hospitals to smaller rural community hospitals located within another province.

A careful investigation of the mobility trend over time indicates that this might be due to the high-variability observed pre-intervention across units, and accounting for this variability, the rate of increase in mobility appears to be higher during the intervention time. A slight decrease in post-intervention is expected after the intervention activities are no longer as prominent and as sites begin to embed sustainability processes into their work processes. Despite considering sustainability both at the beginning and end of implementation, there were issues with sustainability within the participating community hospitals post-intervention. In an attempt to address this, the central MOVE team held end of study conference calls with the local implementation teams at each site to discuss strategies to improve sustainability. The effects of interventions tended to wane over time. The NHS model of sustainability attempts to address this by considering process, staff and organizational factors [26]. Based on the NHS Sustainability survey findings, consideration needs to be given to the organizational fit and infrastructure, including engagement of senior and clinical leadership, and staff knowledge, skills, attitudes and involvement. Additionally, change processes, such as the credibility of the associated evidence, the adaptability of the improvement process and the broad benefits beyond just those for the patient all play a role [32, 33].. The implementation team members that were interviewed identified the importance of having strong champions, involving and educating front-line staff, and embedding the practice change into work processes and culture as all being key to sustainbility.

In addition to the issues with sustainability discussed above, potential limitations to the MOVE intervention have been highlighted elsewhere [16], but include that mobility audits were not conducted continuously and did not measure how far patients ambulated during their day, external factors that might impact length of stay were not considered, and that the primary outcome was the behaviour change (mobilization) and not clinical outcomes associated with immobility such as falls, delirium and functional decline. However, the fact that we have demonstrated increased mobility rates and a non-signficant decreasing trend in hospital length of stay in a number of different hosptials across two different provincial healthcare systems suggests that MOVE is an effective and spreadable change intervention despite its potential limitations.

## Conclusions

Hospitalization can be a pivotal event in an older adult’s life. How care is delivered in hospital can have long-term impact to both the older adult and the health care system. Despite the benefits of early mobilization [9–11, 15], many older adults continue to spend most of their hospital stay in bed [10, 12]. This study demonstrates that a simple adaptable change intervention targeted at improving mobilization among older hospitalized patients can be implemented across a broad array of clinical settings, and within different health care organizations and structures, without the addition of new clinical resources.

## Supplementary information

**Additional file 1.** Template for Intervention Description and Replication (TIDieR) checklist.

**Additional file 2.** MOVE AB: Timeline of phases, activities and data collection.

**Additional file 3.** Pre- and Post- Intervention NHS Sustainability Factor Level Results.

**Additional file 4.** Audit Tool.

**Additional file 5.** Patient and Caregiver Exit Survey.

**Additional file 6.** Staff Exit Survey.

**Additional file 7.** Implementation Activities Delivered.

**Additional file 8.** Staff and Patient Exit Survey Participants by Site.

**Additional file 9.** Implementation Team Interview Participants by Site.

## Data Availability

Study data is maintained by the authors. Please send all requests for study data to Dr. Sharon Straus (Sharon.Straus@utoronto.ca).

## Abbreviations

ITS: Interrupted time series
LOS: Length of stay
MOVE AB: Mobilization of Vulnerable Elders (MOVE) in Alberta
