# Supplementary figures and images for: Scaling-up implementation in community hospitals: a multisite interrupted time series design of the Mobilization of Vulnerable Elders (MOVE) program in Alberta
Source: BMC Geriatr. 2019 Oct 25;19:288. doi: 10.1186/s12877-019-1311-z (PMC6815022; doi:10.1186/s12877-019-1311-z)

**Additional file 2: MOVE Program Timeline**

**
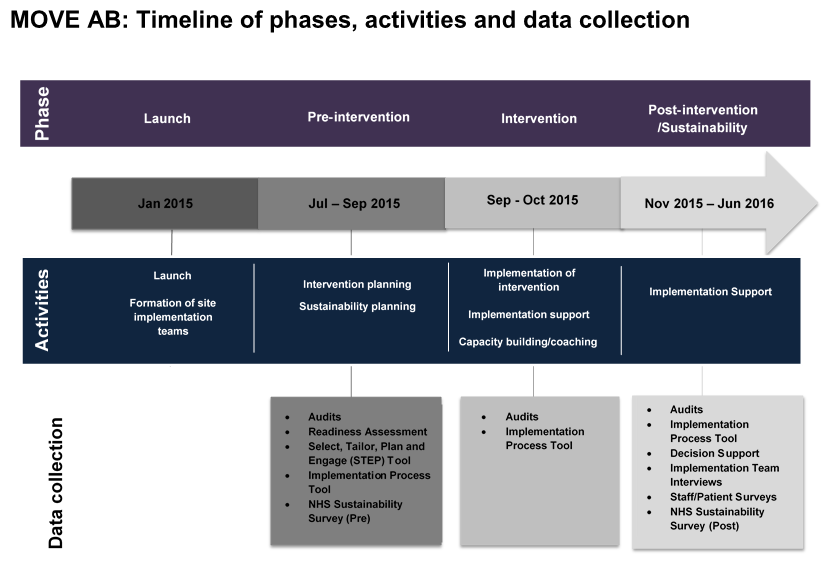
**

Supplement: Supplementary file 2 — Additional file 2. MOVE AB: Timeline of phases, activities and data collection. [file 12877_2019_1311_MOESM2_ESM.docx]
