# Supplementary material for: Scaling-up implementation in community hospitals: a multisite interrupted time series design of the Mobilization of Vulnerable Elders (MOVE) program in Alberta
Source: BMC Geriatr. 2019 Oct 25;19:288. doi: 10.1186/s12877-019-1311-z (PMC6815022; doi:10.1186/s12877-019-1311-z)
Supplement: Supplementary file 3 — Additional file 3. Pre- and Post- Intervention NHS Sustainability Factor Level Results. [file 12877_2019_1311_MOESM3_ESM.docx]

**Additional file 3: Pre- and Post-Intervention NHS Sustainability Factor Level Results Across Sites (n=4) ***

| **Factor Level** | **Max Score** | **Site A** | | **Site B** | | **Site C** | | **Site D** | |
| --- | --- | --- | --- | --- | --- | --- | --- | --- | --- |
|  |  | **Pre** | **Post** | **Pre** | **Post** | **Pre** | **Post** | **Pre** | **Post** |
| **Process** | **31.1** | **18.1** | **18.0** | **5.5** | **6.5** | **21.3** | **12.6** | **27.9** | **27.9** |
| **Benefits beyond helping your patients**  *Ensuring that the change does bring benefits to staff, patients, and the organization* | 8.5 | 4.7 | 4.7 | 0 | 0 | 4.7 | 4.7 | 8.5 | 8,5 |
| **Credibility of the evidence**  *Ensuring that there is evidence that the change will produce benefits that are obvious to all key stakeholders.* | 9.1 | 3.1 | 6.3 | 3.1 | 3.1 | 6.3 | 3.1 | 9.1 | 9.1 |
| **Adaptability**  *Ensuring that the improvement can continue in the face of ongoing changes in staff, leadership, organization structures, etc.* | 7.0 | 7.0 | 7.0 | 2.4 | 3.4 | 7.0 | 2.4 | 7.0 | 7.0 |
| **Monitoring progress**  *Ensuring that you have a system in place to continually and effectively monitor the progress of change.* | 6.5 | 3.3 | 0 | 0 | 0 | 3.3 | 2.4 | 3.3 | 3.3 |
| **Staff** | **52.4** | **23.8** | **32.1** | **17.5** | **32.1** | **52.4** | **21.7** | **20.5** | **25.0** |
| **Involvement**  *Ensuring that key staff at all levels who are affected by the change, can contribute are involved from the outset and trained in any new skills needed.* | 11.4 | 6.3 | 6.3 | 0 | 6.3 | 11.4 | 4.9 | 0 | 4.9 |
| **Behaviours**  *Reducing skepticism by increasing belief in the change and helping staff to feel empowered in their work.* | 11.0 | 5.1 | 5.1 | 5.1 | 5.1 | 11.0 | 5.1 | 0 | 5.1 |
| **Senior leadership engagement**  *Engaging senior leaders and encouraging them to interact with staff and take responsibility for sustaining change.* | 15.0 | 5.7 | 5.7 | 5.7 | 5.7 | 15.0 | 6.2 | 15.0 | 0 |
| **Clinical Leadership Engagement**  *Engaging clinicians and encouraging them to be involved in the project's success.* | 15.0 | 6.7 | 15.0 | 6.7 | 15.0 | 7.0 | 5.5 | 5.5 | 15.0 |
| **Organization** | **16.5** | **3.3** | **12.8** | **0** | **0** | **11.4** | **6.6** | **12.8** | **7.7** |
| **Fit with organization's strategic aims and culture**  *Ensuring that there is synergy between the improvement and organizational goals and vision.* | 7.0 | 3.3 | 3.3 | 0 | 0 | 7.0 | 3.3 | 3.3 | 3.3 |
| **Infrastructure for sustainability**  *Ensuring the improvement effort is supported during and beyond the formal life of the project.* | 9.5 | 0 | 9.5 | 0 | 0 | 4.4 | 3.3 | 9.5 | 4.4 |
| Total Sustainability Score | **100.0** | **45.2** | **62.9** | **23.0** | **38.6** | **85.1** | **40.9** | **61.2** | **60.6** |

*Survey items and factor level was developed by Maher L, Gustafson D, Evans A. Sustainability model and guide. NHS Institute for Innovation and Improvement. 2007. Retrieved from: <https://webarchive.nationalarchives.gov.uk/20160805122935/http://www.nhsiq.nhs.uk/media/2757778/nhs_sustainability_model_-_february_2010_1_.pdf>
