# Supplementary material for: Scaling-up implementation in community hospitals: a multisite interrupted time series design of the Mobilization of Vulnerable Elders (MOVE) program in Alberta
Source: BMC Geriatr. 2019 Oct 25;19:288. doi: 10.1186/s12877-019-1311-z (PMC6815022; doi:10.1186/s12877-019-1311-z)
Supplement: Supplementary file 5 — Additional file 5. Patient and Caregiver Exit Survey. [file 12877_2019_1311_MOESM5_ESM.docx]

**Additional file 5: Patient and Caregiver Exit Survey**

**Patient and Caregiver Survey**

**Part 1: Demographics**

1. Please indicate if you are one of the following:
2. Patient
3. Family Member/Friend of a patient
4. Approximately how long is your (or your family member’s/friend’s) anticipated stay in hospital?
5. Less than 3 days
6. 3 days to 1 week
7. 1 to 3 weeks
8. 3 weeks or more

**Part 2: Mobilization**

1a. Which of the following activities did you or the patient participate in, or were encouraged to participate in, during the hospital stay:

| Patient Activity | Participation  (Check most appropriate box) | | | |
| --- | --- | --- | --- | --- |
|  | Participated | Encouraged to participate | Did not participate | Unsure |
| [Activity 1] |  |  |  |  |
| [Activity 2] |  |  |  |  |
|  |  |  |  |  |

1b. Of the activities you participated in, please let us know which one you found to be most helpful, and which one was the least helpful.

Most helpful: ______________________ Least helpful: ________________________

2. If you read, used or participated in any of these education activities, please rate how effective you think it was in **increasing your knowledge about the importance of moving while in the hospital,** on a scale of 1(not effective) to 5 (extremely effective). If you did not read, use or participate in any activity listed, please select “N/A”.

1 2 3 4 5 N/A

Not Extremely

Effective Effective

3. If you read, used or participated in any of these educational activities, please rate how effective you think it was in **helping you to move while you, or the patient, were in the hospital,** on a scale of 1(not effective) to 5 (extremely effective). If you did not read, use or participate in any activity listed below, please select “N/A”.

1 2 3 4 5 N/A

Not Extremely

Effective Effective

4. How often do you remember hospital staff (e.g., Nurse, Physician, Physiotherapist, Occupational Therapist, Volunteer, Other) approaching you or the patient to encourage you or the patient to move (i.e., get out of bed, go for a walk, sit in a chair), during your hospital stay?

Never Rarely Sometimes Frequently Always

5. If they did approach you, which type of hospital staff approached you or the patient to encourage you or the patient to move, during the hospital stay?

Please circle all that apply:

Nurse Physician Physiotherapist

Occupational Therapist Volunteer Other: ________________________

**Part 3: Reflection**

1. Please provide any additional comments you may have on mobilization during your hospital stay.

2. Please provide suggestions you may have for improving mobilization of patients such as yourself or your loved one, based on your own or your loved one’s hospital experience.
