# Supplementary material for: Scaling-up implementation in community hospitals: a multisite interrupted time series design of the Mobilization of Vulnerable Elders (MOVE) program in Alberta
Source: BMC Geriatr. 2019 Oct 25;19:288. doi: 10.1186/s12877-019-1311-z (PMC6815022; doi:10.1186/s12877-019-1311-z)
Supplement: Supplementary file 6 — Additional file 6. Staff Exit Survey. [file 12877_2019_1311_MOESM6_ESM.docx]

**Additional file 6: Staff Exit Survey**

**Part 1: Education Intervention**

Scales to use:

1(not effective), 2 (somewhat effective), 3 (not sure), 4 (effective), 5 (extremely effective).

1(not appropriate), 2 (somewhat appropriate), 3 (not sure), 4 (appropriate), 5 (extremely appropriate)

1. A) Please rate the following education activities for their **effectiveness in increasing your knowledge on early mobilization** on a scale of 1(not effective) to 5 (extremely effective). If you did not participate in, use, or observe an activity listed below, please select “N/A”.

*[list of all activities the unit selected as part of the intervention. This is unknown at this point in time.]*

B) Please elaborate on your responses for activities you rated a 1 or 5 (i.e. why you gave these activities a score of 1 or 5).

1. A) Please rate the following education activities for their **effectiveness in changing staff support for early mobilization** on a scale of 1(not effective) to 5 (extremely effective). If you did not participate in, use, or observe an activity listed below, please select “N/A”.

*[list of all activities the unit selected as part of the intervention. This is unknown at this point in time.]*

B) Please elaborate on your responses for activities you rated a 1 or 5 (i.e. why you gave these activities a score of 1 or 5).

1. A) Please rate the following education activities for their **effectiveness in changing patient/family knowledge of early mobilization** on a scale of 1(not effective) to 5 (extremely effective). If you are not aware of the use of these activities on your unit, please select “N/A”.

*[list of all activities the unit selected as part of the intervention. This is unknown at this point in time.]*

B) Please elaborate on your responses for activities you rated a 1 or 5 (i.e. why you gave these activities a score of 1 or 5).

1. A) Please rate the following education activities for their **effectiveness in changing patient/family support for early mobilization** on a scale of 1(not effective) to 5 (extremely effective). If you are not aware of the use of these activities on your unit, please select “N/A”.

*[list of all activities the unit selected as part of the intervention. This is unknown at this point in time.]*

B) In the space below, please elaborate on your responses for activities you rated a 1 or 5 (i.e. why you gave these activities a score of 1 or 5).

1. A) Please rate the following education activities for their **appropriateness for implementation on your unit** on a scale of 1(not appropriate) to 5 (extremely appropriate):

*[list of all activities the unit selected as part of the intervention. This is unknown at this point in time.]*

B) In the space below, please elaborate on your responses for activities you rated a 1 or 5 (i.e. why you gave these activities a score of 1 or 5).

**Part 2: Mobilization**

1. A) For each of the professions listed below, please circle the effectiveness of MOVE AB in enhancing that profession’s role in early mobilization on a scale of 1(not effective) to 5 (extremely effective).
   1. Physician: 1 2 3 4 5
   2. RN: 1 2 3 4 5
   3. RN (student or CA): 1 2 3 4 5
   4. OT: 1 2 3 4 5
   5. PT: 1 2 3 4 5
   6. Other Allied Health Professional (specify): 1 2 3 4 5
   7. Administrator: 1 2 3 4 5

B) In the space below, please elaborate on your responses you rated a 1 or 5 (i.e. why you gave these a score of 1 or 5).

1. In the space below, please describe the key barriers to mobilizing patients on your unit.
2. In the space below, please describe the key facilitators to mobilizing patients on your unit.

**Part 3: Reflection**

1. In the space below, please provide suggestions on what could have been done differently to enhance the implementation of MOVE AB.
2. In the space below, please provide suggestions on how to increase the sustainability of the MOVE AB intervention.

**Part 4: Climate of Mobilization**

Please rate the extent to which you agree with each of the following statements on a scale from 0 (not at all) to 4 (to a very great extent).

1. I like to use early mobilization practices to help my patients.
2. I am willing to try mobilization practices even if I have to follow protocols/ evidence-based practices for appropriate mobilization initiation, assessment and/or documentation.
3. I know better than academic researchers how to care for my patients.
4. I am willing to use new and different types of protocols/ practices developed by researchers.
5. Research based protocols/ practices are not clinically useful.
6. Clinical experience is more important than using protocols/ evidence-based practices for early mobilization practices (e.g. appropriate mobilization initiation, assessment and/or documentation.)
7. I would not use protocols/ evidence-based practices for early mobilization such as appropriate mobilization initiation, assessment and/or documentation.
8. I would try early mobilization of my patients even if it was very different from what I am used to doing in my role.

If you received training in early mobilization of patients (e.g. how to mobilize, when to mobilize, etc.) how likely would you be to adopt it if:

1. It was intuitively appealing?
2. It “made sense” to you?
3. It was required by your supervisor/manager?
4. It was required by your hospital/institution?
5. It was required by your province?
6. It was being used by colleagues who were happy with it?
7. You felt you had enough training to mobilize your patients correctly?

**Part 5: Demographics**

1. Profession: *please indicate your role:*
   1. Physician
   2. RN
   3. RN (student)
   4. OT
   5. PT
   6. Other Allied Health Professional (specify)
   7. Administrator
2. Years of practice
   1. Less than 1 year
   2. 1 to 5 years
   3. 5 to 10 years
   4. 10 to 20 years
   5. 20+ years
3. On which hospital unit at [insert hospital name here] do you work?
   1. [insert unit name]
   2. [insert unit name]
