# Supplementary material for: Scaling-up implementation in community hospitals: a multisite interrupted time series design of the Mobilization of Vulnerable Elders (MOVE) program in Alberta
Source: BMC Geriatr. 2019 Oct 25;19:288. doi: 10.1186/s12877-019-1311-z (PMC6815022; doi:10.1186/s12877-019-1311-z)
Supplement: Supplementary file 7 — Additional file 7. Implementation Activities Delivered. [file 12877_2019_1311_MOESM7_ESM.docx]

**Additional file 7: Implementation Activities Delivered**

|  |  |  | **Site** | | | |
| --- | --- | --- | --- | --- | --- | --- |
| **Type** | **Activity** | **Description** | Site A | Site B | Site C | Site D |
| Educational Meetings (In-person or electronic) | Interprofessional staff education module^a^ | Classroom-based module to help prepare staff for a change in clinical practice related to mobilization by facilitating discussion and acknowledging potential challenges and barriers in a group setting | 🗸 | 🗸 | 🗸 | 🗸 |
|  | MOVE electronic module^a^ | Electronic module used to quickly reach a large number of staff, with content similar to that of the classroom-based module |  |  |  |  |
|  | MOVE ON senior-friendly hospitals module^a^ | Electronic module for interprofessional hospital staff to review the risks of hospitalization for older adults, reflect on the effects of ageism and stereotyping of older adults, and review the needs of older adults |  |  |  |  |
|  | One- on- One Coaching^b^ | Education Coordinators provided bedside coaching with staff to walk through potential ways to mobilize patients in different scenarios | 🗸 |  | 🗸 | 🗸 |
| Staff Coaching Tools | Technical Assistance | Ongoing implementation support with staff to assess context and improve efforts for implementation, sustainability and scale-up. | 🗸 | 🗸 | 🗸 | 🗸 |
|  | Review of ABCs Educational  Tool | Standard of care for mobility | 🗸 | 🗸 |  |  |
|  | Documentation practices | Encourages proper documentation of patients’ mobility status | 🗸 | 🗸 | 🗸 | 🗸 |
|  | Transfer techniques and ergonomics education sessions | Presents techniques such as ‘roll’, ‘lie to sit’, and ‘sit to stand’ | 🗸 | 🗸 |  |  |
|  | Natural opportunities | Encourages creative ways to incorporate mobility into everyday practice | 🗸 | 🗸 |  | 🗸 |
| Distribution of printed educational materials | Mobility algorithm | Tool to help staff assess each patients’ mobility status and to  aid in communicating patients’ mobility status through the use of (ABC) letters to identify mobility level | 🗸 | 🗸 |  |  |
|  | Hazards of immobility poster | Educational poster for hospital staff | 🗸 | 🗸 | 🗸 | 🗸 |
|  | Benefits of getting out of bed while in hospital poster | Educational poster for patients/family members | 🗸 | 🗸 | 🗸 | 🗸 |
|  | Keep moving pamphlet | Educational pamphlet for patients/family members | 🗸 | 🗸 | 🗸 | 🗸 |
| Reminders | Commercial breaks | One-minute musical interludes during multidisciplinary or bullet rounds with messages to encourage mobilization |  |  |  |  |
| Communication and case discussion | Mobility Champions | Role-models and/or mentors who encourage, carry out and facilitate the safe and effective mobilization of patients by promoting staff involvement in the routine mobilizing of patients. | 🗸 | 🗸 | 🗸 | 🗸 |
|  | Huddles | Quick stand-up staff meetings to discuss progress of intervention, and share successes and challenges | 🗸 | 🗸 | 🗸 | 🗸 |
| Educational Exhibits | MOVE ON Fair | Series of 8 stations set up in a common area on at least 2 separate days, where staff members can learn about: MOVE ON, documentation practices, myths about mobilization, and other relevant aspects of the project |  |  |  |  |

^a^- Although staff education was mandatory, the mode of education was at the discretion of each site.

^b^- Knowledge-to-practice coaching is delivered at the point of care. It can be used to engage staff on an individual basis to support mobility

coaching by the bedside and relates knowledge and skills directly to patients that the staff member is caring for that day
