# Supplementary material for: Scaling-up implementation in community hospitals: a multisite interrupted time series design of the Mobilization of Vulnerable Elders (MOVE) program in Alberta
Source: BMC Geriatr. 2019 Oct 25;19:288. doi: 10.1186/s12877-019-1311-z (PMC6815022; doi:10.1186/s12877-019-1311-z)
Supplement: Supplementary file 8 — Additional file 8. Staff and Patient Exit Survey Participants by Site. [file 12877_2019_1311_MOESM8_ESM.docx]

**Additional file 8: Staff and Patient Exit Survey Participants by Site**

|  | | **Site A** | **Site B** | **Site C^1^** | **Site D^2^** |
| --- | --- | --- | --- | --- | --- |
| **Staff** (N = 29) | Physicians (n = 5) | 5 | - | - | - |
|  | Nursing (n = 8) | 2 | 1 | 5 | - |
|  | Administrators (n = 2) | 1 | - | 1 | - |
|  | Physiotherapist (n = 2) | 2 | - | - | - |
|  | Other Allied Health Professionals (n = 12) | 7 | 4 | 1 | - |
| **Patient/Family/** **Caregiver**  (N = 45) | Patient (n = 39) | 19 | 13 | - | 7 |
|  | Caregiver (n = 3) | - | 1 | - | 2 |
|  | Unidentified (n = 3) | 2 | 1 | - | - |

^1^Site C did not complete Patient/Family/Caregiver exit surveys

^2^Site D did not complete staff exit surveys
