# Supplementary material for: Scaling-up implementation in community hospitals: a multisite interrupted time series design of the Mobilization of Vulnerable Elders (MOVE) program in Alberta
Source: BMC Geriatr. 2019 Oct 25;19:288. doi: 10.1186/s12877-019-1311-z (PMC6815022; doi:10.1186/s12877-019-1311-z)
Supplement: Supplementary file 9 — Additional file 9. Implementation Team Interview Participants by Site. [file 12877_2019_1311_MOESM9_ESM.docx]

**Additional file 9 Implementation Team Interview Participants by Site**

| **Staff**  (N = 9) | **Site A** | **Site B** | **Site C** | **Site D** |
| --- | --- | --- | --- | --- |
| Unit Manager (n = 4) | 2 | 1 | - | 1 |
| Physiotherapist (n = 1) | 1 | - | - | - |
| Nurse Educator (n = 3) | - | 1 | 1 | 1 |
| Other (n = 1) | 1 | - | - | - |
