# Supplementary material for: Scaling-up implementation in community hospitals: a multisite interrupted time series design of the Mobilization of Vulnerable Elders (MOVE) program in Alberta
Source: BMC Geriatr. 2019 Oct 25;19:288. doi: 10.1186/s12877-019-1311-z (PMC6815022; doi:10.1186/s12877-019-1311-z)
Supplement: Supplementary file 9 [file 12877_2019_1311_MOESM4_ESM.docx]

move
Promoting Mobilization

Site Code: Date: / /2015 Observer Initials: Unit:
Phase (Check): ☐ *Pre* ☐ During Education ☐ Post

Appendix 4: Audit Tool

Patient Status	Type of mobility **directly observed**	Chair Available?
0 = On Ward<br>1 = Test (off ward)<br>2 = Procedure (off ward)<br>3 = Physiotherapy/Rehab (off ward)<br>4 = Deceased<br>5 = Other<br>6 = Discharged or Transferred<br>7 = Off Ward (use only if other codes do not apply)<br>8 = Palliative Patient<br>99 = Not Documented	0 = Laying In bed (HOB ≤ 30°)<br>1 = Laying in bed (HOB > 30°)<br>2 = Sitting in bed, legs off side/dangling<br>3 = Sitting in chair<br>4 = Standing/walking with assistance<br>5 = Standing/walking supervised<br>6 = Standing/walking independently with or without walking aid (e.g. cane, walker, etc.)<br>7 = Not in room and not observed doing any of the above<br>8 = In washroom – status not known<br>9 = Curtain/door closed – status not known<br>10 = Bed rest (use only for surgery, orthopedic, or trauma units)<br>99 = Not Documented	0 = No<br>1 = Yes<br>99 = Not Documented<br>**Patient on Isolation?**<br>0 = No<br>1 = Yes<br>99 = Not documented

ID	Bed#	Age	AM				Lunch				PM			
			Patient Status	Mobility Observed	Chair?	Isolation	Patient Status	Mobility Observed	Chair?	Isolation	Patient Status	Mobility Observed	Chair?	Isolation
:														
↓														
